# Evaluating a targeted selective speech, language, and communication intervention at scale – Protocol for the Happy Talk cluster randomised controlled trial.

**DOI:** 10.12688/hrbopenres.13973.3

**Published:** 2025-01-31

**Authors:** Pauline Frizelle, Aoife O'Shea, Aileen Murphy, Darren Dahly, Cristina McKean

**Affiliations:** 1 Department of Speech and Hearing Sciences, School of Clinical Therapies, University College Cork, Cork, Ireland; 2 Speech and Language Therapy Department, Health Services Executive, Cork, Ireland; 3 Department of Economics, University College Cork, Cork, Ireland; 4 School of Public Health, University College Cork, Cork, Ireland; 5 Department of Education, Oxford University, Oxford, UK

**Keywords:** Language development, Language development disorders, Public Health, Early intervention, Outcome assessment, Child development, Cluster randomized controlled trial.

## Abstract

**Background:**

In areas of social disadvantage up to 40–50% of children enter preschool with speech and language skills significantly poorer than would be expected for their age. The Happy Talk trial tests if a community embedded, targeted selective speech and language programme that simultaneously engages with parents and early childhood educators, (1) improves language outcomes in children aged between 2 years 10 months and 6 years and (2) is cost effective for the health care system.

**Method:**

The Happy Talk trial is a large scale cluster randomised trial of a 12-week manualised intervention delivered in pre/school settings serving socially disadvantaged communities, in Ireland. Seventy-two clusters will receive the intervention (12 participants per cluster). Parents and pre/school staff engage in group training and coaching in the form of 12 1-hour sessions for parents and four staff workshops, over the course of the pre/school year. Training/coaching includes core interaction skills (modelling, expanding, balancing questions and comments), early literacy and phonological awareness. Blinded assessments pre- and immediately post-intervention and at 6 months follow up, will measure the primary outcomes of children’s receptive and expressive language and functional impact, and secondary outcomes of quality of life. Parental responsiveness and educator-child interactions will also be evaluated.

**Discussion:**

This robust study evaluates a public health approach to the delivery of speech language and communication intervention in the ‘real world’ in the community, which focuses on prevention and equity of access. Pilot work indicates that the programme is feasible, acceptable to parents and staff, cost effective, and suitable for implementation at scale. The trial includes a process evaluation, a well-developed economic evaluation and the outcomes are directly relevant to children, families and educators. This work has the potential to improve the long-term outcomes and life chances of people living in social disadvantage.

**Trial registration:**

clinicaltrials.gov NCT06460090

**Trial Management:**

There is a formal governance structure to oversee the conduct and running of the trial, consisting of a trial management group and a steering committee. More details on the composition, roles and responsibilities of each committee can be found in the supplemental material.

## Background

Language is an important health outcome because it is a key predictor of quality of life ( Field, 2010), social-emotional and behaviour difficulties ( Qi & Kaiser, 2003) as well as mental health and employment outcomes ( Clegg *et al.*, 2005; Law *et al.*, 2009). If language difficulties are untreated, there are long-term health, educational and societal consequences ( Law *et al.*, 2009), which in turn place a significant economic burden on society. Moreover, in recent years the effects of these difficulties have been further exacerbated by the COVID-19 pandemic ( Erbay & Tarman, 2022; Nelson *et al.*, 2021).

### Language in the context of social disadvantage

Although rates of language difficulty are reported to vary considerably (e.g. Norbury *et al.*, 2016; Tomblin *et al.*, 1997), literature converges on the view that children from disadvantaged backgrounds have disproportionately higher rates of language difficulty than their more affluent peers ( McKean *et al.*, 2018; Morgan *et al.*, 2015; Nelson *et al.*, 2011). This higher rate of language difficulty has been documented in children as early as 18 months ( Fernald *et al.*, 2013; McGillion *et al.*, 2017), and without intervention, does not remediate as children progress through primary and secondary schooling ( Duncan & Magnuson, 2011). Children with such persisting difficulties being eligible for a diagnosis of DLD. In the US, Nelson and colleagues reported as many as 65% of children living in social disadvantage met the criteria for a clinically significant language impairment ( Nelson *et al.*, 2011). While in the UK, Locke and colleagues (2002) reported a prevalence of 50% of children living in significant deprivation, starting preschool with lower levels of language than would be expected for their age. Similarly, Law *et al.* (2011) reported an almost 40% prevalence rate in slightly older Scottish children (5 – 12 years), living in considerable social disadvantage. Applying these figures to the 30% of children living in poverty in the UK would indicate that there are between 1.7 and 2.15 million children with language skills not at their expected level and not attributable to a specific diagnosis. Moreover, a teacher report published by Speech and Language UK (2023), indicated a 26% increase in children with language difficulties in the previous two years.

Historically, the association between social disadvantage and lower language levels was primarily attributed to children experiencing fewer examples of rich language input ( Hart & Risley, 1995), fewer opportunities for quality caregiver–child interactions, and parenting styles that are less responsive to children’s interests ( Landry *et al.*, 2001). However, while these factors do mediate children’s language development ( Morgan *et al.*, 2015), more recent research has highlighted the complexity and multiplicity of components at play in attempting to explain the relationship between poor language and social disadvantage ( Law *et al.*, 2019). In particular the issue of access and engagement with services has been highlighted as problematic ( Moore *et al.*, 2015), and a key consideration in the design and implementation of interventions ( McKean & Reilly, 2023; Reilly & McKean, 2023).

Given the high rates of language difficulty and the consequences that these difficulties have on children’s lives, coupled with issues of access, providing effective and efficient interventions that are accessible for all groups is a clear priority, to achieve improved health outcomes in socially disadvantaged populations. One of the best ways to ensure access and participation and therefore to increase the impact and reach of early language interventions, is through interventions that are universal, preventative, and embedded within community based organisations with which parents are already engaging.

### Early childhood educator professional development programmes

One approach to support children’s development in naturalistic community based environments is through the use of early childhood educator professional development programmes. Programmes have been developed with the aim of training early childhood educators in evidence-based techniques and practices that create language rich environments and encourage responsive styles of interaction. By enhancing educators’ knowledge and optimising their use of language promoting techniques, it is hypothesized to have a cascading effect, improving children’s language outcomes. This is supported by the empirical literature which shows that high quality early childhood education can mitigate the effects of poverty in relation to preschool language ( Lim *et al.*, 2022; Marulis & Neuman, 2013; Schachter, 2015). In broad terms, early childhood educator professional development programmes tend to have a primary focus, either on educator–child responsive interactions, or on literacy. Consequently, outcomes are very variable and findings in relation to child language outcomes are inconsistent. With respect to professional development interventions focusing on educator-child responsive interactions, Eadie *et al.* (2019) and Cabell *et al.* (2011) reported no effects on child language outcomes. In contrast, Yazejian *et al.* (2020) and Piasta *et al.* (2012) reported positive (albeit modest) effects in relation to auditory language skills and linguistic productivity respectively. Professional development programmes with a literacy focus tend to report more consistent positive findings, particularly in relation to the specific areas being targeted e.g. letter knowledge, concepts about print and phonological awareness ( Markussen-Brown *et al.*, 2017; Powell *et al.*, 2010). However, positive impact of these programmes on broader language outcomes, such as vocabulary, is less consistent ( Landry *et al.*, 2009; Markussen-Brown *et al.*, 2017; Wasik & Hindman (2011). Nonetheless, studies do indicate a positive impact on educators, such as an increase in the instructional quality of their teaching ( Bowne *et al.*, 2016; Eadie *et al.*, 2019) and the use of sensitive/ responsive strategies ( Landry *et al.*, 2017). There are many reasons that may explain why these effects do not seem to translate into positive gains for children’s language outcomes, not least the timing of outcome measures and the absence of follow up /longitudinal data. It may also be that positive changes in one environment are not sufficiently intense or consistent to be reflected in children’s language skills either in the short- or long-term. One potential solution to this is to engage with parents with the aim of increasing parent responsiveness and support for language in children’s home environments. 

### Parent-mediated intervention programmes

Parent-mediated interventions are preventative approaches, frequently used with socially disadvantaged families which aim to train parents in the use of responsive and contingent interactions with their child. These interventions are based on the premise that parent-child interactions and the activities that parents and children share together offer opportunities for promoting early language learning. Parents are taught strategies to interact responsively, to support mutual engagement with their child and provide a higher quantity of verbal input, matched to their child’s interests and developmental level ( Golinkoff *et al.*, 2015; Hoff, 2006; Rowe & Snow, 2020). Subsequently, they are encouraged to use these interactive and linguistic strategies in everyday routines such as playtime, shared book reading etc. This approach is supported by the empirical literature, in that parent-child interactions with specific qualities (responsive, contingent and developmentally appropriate) are associated with better language outcomes ( Levickis *et al.*, 2023; Madigan *et al.*, 2019). However, although usually greater for children from disadvantaged backgrounds, effect sizes are often modest. Based on two meta-analyses of parent behaviour and child language, Madigan and colleagues (2019) reported a weak association between sensitive responsive parenting and child language, with effect sizes stronger in those with low SES. In a more recent meta-analysis Jeong *et al.* (2021) also reported relatively modest positive effects of parenting interventions on children’s language development, standard mean difference = 0.28, 95% CI: 0.18 to 0.37, *p* < 0.001), again with significantly greater effects on children from low- compared to high-income countries. In an additional meta-analysis Roberts *et al.* (2019) explored the association between parent training (naturalistic and dialogic reading) and child language development and reported moderate effect sizes for children at risk, in relation to receptive language (mean Hedges g [SE] = 0.28 [0.15]) and engagement outcomes (mean Hedges g [SE] = 0.36 [0.17]). Overall, in the short term, pooled effect sizes for parent mediated interventions for those living in social disadvantage are at best moderate and it would seem that, similar to professional development programmes, this approach alone is not sufficient to support children living in these circumstances.

### Interventions engaging with parents and early childhood educators

Despite relatively modest effects for programmes that are aimed solely at either early childhood educators or parents/caregivers, studies of programmes that simultaneously engage with both groups are relatively scarce ( Greenwood *et al.*, 2020), particularly for those with child language outcome measurements. By providing an intervention in more than one environment, to two sets of people who spend a significant amount of time with the child, the expectation is that the exposure to responsive interactions and language-promoting strategies will increase. This, in turn, is hypothesized to increase the language gains for the child. Findings by Frizelle *et al.* (2021a) (Happy Talk); Gibbard *et al.* (2024) (Enhanced parent based intervention) and Stevens *et al.* (2019), (Abecedarian approach) support this hypothesis. Frizelle *et al.* (2021a) carried out pilot effectiveness study and found that implementing a community-based speech language and communication programme (Happy Talk) that simultaneously engaged with parents and early childhood educators resulted in large effects (.6SD) on comprehension and moderate effects (.46SD) on a composite language measure (comprehension and expression), for children from areas of social disadvantage. In addition, the pilot trial indicated cost effectiveness ( Frizelle *et al.*, 2021b), as well as feasibility and acceptability to parents and preschool staff, making Happy Talk suitable for implementation at scale. In an RCT, Gibbard *et al.* (2024) compared a parent mediated clinic-based intervention to one that included early years educators in a local children’s centre and reported greater effect sizes for almost all outcomes when a more integrated model of intervention delivery was used.

In contrast, other studies have reported no additional child language benefits to carrying out an intervention in two environments (home and school) over one (home alone) ( Hargrave & Sénéchal, 2000; Landry *et al.*, 2017; Lonigan & Whitehurst, 1998). Therefore, while there is some evidence of an increased benefit to simultaneously engaging with parents/caregivers at home and early years educators in pre/school, the evidence is not conclusive. 

In addition, whether parent-mediated, early childhood educator focused, or both, many intervention studies are small with limited follow up; they tend to be ‘efficacy’ trials carried out in very controlled environments rather than ‘real world’ effectiveness studies based in the community; they are not pre-specified in a protocol and there are few replications. Moreover, there is a need for more longitudinal research as well as studies specifically focused on scaling-up interventions, including features such as community readiness and capacity, program acceptability and cost. The current work aims to build on the Happy Talk pilot trial by scaling up to a full definitive effectiveness trial carried out in the community, including follow up outcome measures to look at the longer term impact of the programme; a process evaluation and a societal cost benefit analysis at a larger scale. In doing so the work will address the aforementioned issues above.

Objectives/ Hypotheses

The Happy Talk trial aims to answer 3 specific research questions.

1. Does Happy Talk, a targeted selective intervention focused on increasing parent and early educator responsive interaction, improve language and quality of-life (QoL) outcomes in socially disadvantaged preschool and young school-aged children, compared to usual care?
2. Does Happy Talk enhance language promoting behaviours /responsiveness in home and pre/school contexts?
3. Is Happy Talk cost effective for the health care system compared to usual care?
4. What programme features support successful real-world application of ‘Happy Talk’ and how do contextual factors influence Happy Talk implementation/ outcomes?

We hypothesize that compared to the control group

1. Children in the intervention group will have better mean scores on
  a. Receptive, expressive and total language score on the standardized language measure, the Preschool Language Scales (PLS-5)
  b. Focus on the Outcomes of Communication under Six (FOCUS)
  c. Paediatric Quality of Life Inventory (PedsQL) and Parent report for Toddlers and Child Health Utility instrument (CHU9D), we have delayed follow up measures to 1 year post to allow time for programme effects to translate into QoL impact.
2. Parents in the intervention group will have higher mean scores on the Maternal Responsive Behaviours Coding Scheme (MRBCS)
3. Early childhood education settings in the intervention group will have higher means scores on the Classroom Assessment Scoring System (CLASS)
4. The intervention will be cost effective for the health care system (measured against common decision thresholds).

## Methods

**Figure 1.**
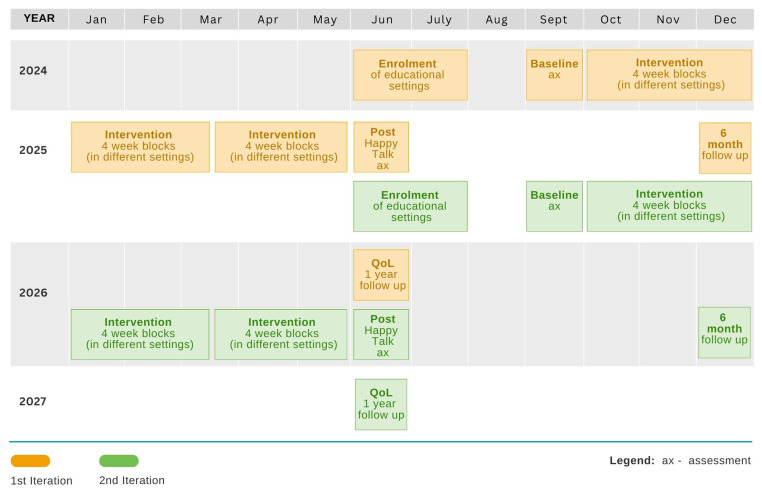
Time schedule for Enrolment, Assessments and Intervention delivery.

The intervention will be evaluated using a cluster-randomized controlled trial taking place over three years. The trial will include two study cycles that each include a period for enrolling settings, baseline assessments, an 8 month intervention period, outcome assessments immediately post-intervention, and additional follow-up measures taken 6 months post-intervention, or 1 year post-intervention for QoL measures. The time schedule for enrolment, intervention delivery and assessments is given in Figure 1. The process evaluation will involve two phases 1) a pre-trial evaluation examining factors which promote parental engagement and partnership between SLTs and educators with the aim of incorporating these into SLT training and future rollouts of the programme and 2) a concurrent evaluation from a realist perspective to examine how the mechanisms underpinning Happy Talk are influenced by the implementation context. A combination of surveys, interviews and focus group methodologies will be used. These will be reported on in more detail elsewhere.

### Inclusion criteria

The HSE in Ireland is divided into nine Community Healthcare Organisations (CHO) each of which has a chief officer who leads the local management team, who in turn govern all specialist services in their area, including primary care SLT. Pre/schools will be targeted for recruitment from 4 CHOs in Ireland for each trial cycle (8 in total) and will be selected based on levels of social disadvantage as indexed by the 2016 Pobal HP Deprivation Index. The index is a method of measuring the relative affluence or disadvantage of geographical area in Ireland. Inclusion criteria for pre/schools are as follows:

- Those falling within the Health Services Executive Community Healthcare Organisation (CHO) area for which support has been offered.
- Those attached to DEIS schools (Delivering Equality of Opportunity in Schools i.e., those including a high concentration of students from socioeconomically disadvantaged backgrounds)
- Child and Family Resource centres (established in Ireland for children from disadvantaged backgrounds).
- Pre/schools who are not in receipt of another early years language intervention programme that is not part of standard care.

While all children of the appropriate age, their parents, and all staff in each intervention setting will be offered the programme, it is not feasible to assess all children in each setting in the time frame before pre/school starts at the beginning of the academic year. To ensure adequate power, of those that consent to be part of the evaluation, 12 participants from each cluster will be randomly chosen for inclusion. We appreciate that those randomly chosen may experience varying degrees of disadvantage. We will collect this demographic information and note individual differences. This variation will also exist within the control settings and therefore should not be a confounding variable.

### Exclusion criteria

Children who are known to have an intellectual disability, or who are non-English speaking will be excluded from the evaluation. Children for whom English is a second language will not be excluded if parents report that they are comfortable completing the outcome measures without the need for interpreters.

### Interventions

***Active intervention***

**Figure 2.**
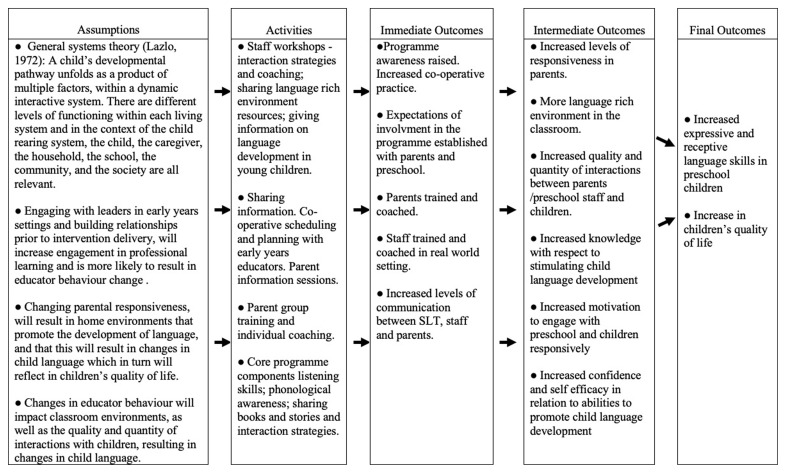
Happy Talk Logic Evaluation Model.

The active intervention under evaluation is Happy Talk, a manualized training and support programme delivered by SLTs to parents and early childhood educators in socially disadvantaged areas. The overall programme aims to support children between 0 and 6 years. However, the focus of this trial is solely on the preschool/Junior infant class programme (children aged between 2;10 and 5,11 years). The programme is informed by general systems theory ( Lazlo, 1972) and is embedded in the preschools, and homes of socially disadvantaged children with the aim of effecting change in parent and educator behaviour (see Figure 2 Happy Talk Logic Evaluation model). 

Parent component: This includes twelve in person 1-hour sessions delivered in 4 week blocks and in two 30-min units, over the three terms of the pre/school year (September–December, January–March and April–June). For the first 30 minutes of each session, parents (mothers, fathers and occasionally other family members) engage in live group training with the SLT in a room within the pre/school. This is followed by 30 minutes of in person coaching, with parents practicing their newly acquired skills with their children in the pre/school, while they are guided and scaffolded by the SLT. The skills targeted in the programme include listening skills; modelling and expanding language; balancing questions and comments; practicing language promoting techniques during free play; phonological awareness (such as rhythm, blending and segmenting syllables); learning new words; pretend play through stories; and bringing books alive.

Pre/school staff component and communication champions: Prior to delivering the staff components of the intervention, therapists engage with each pre/school setting for a period of 2 to 3 weeks to share and schedule how Happy Talk will be delivered over the coming year; discuss maximising parental engagement and attendance; and conduct a parent information session. The aim of this engagement is to begin developing relationships, and to work together as a community prior to inviting parents to participate in the Happy Talk programme. Pre/school staff complete four workshops in total. Workshop 1 takes place in each pre/school before the 12-week parent programme begins. The workshop focuses on the three core interaction skills to be covered with parents in term 1 (modelling, expanding and balancing questions and comments) as well as early literacy and phonological awareness skills. The workshop gives staff the opportunity to practice these skills using a range of pre/school toys and is followed by a 30-min coaching session where staff practice using the skills under the SLT’s supervision, prompting and guidance. Workshops 2 – 4 take place following each 4-week parent intervention block. These workshops include the following core components

- revision of the interaction skills previously outlined
- sharing language rich environment resources
- giving information on language development in young children and on identifying children with speech, language, and communication needs
- sharing speech and language tools that aid with the transition from preschool to school.

The number of staff attending the workshops will vary according to the setting i.e. the number of pre/school rooms/classes within a given setting receiving Happy Talk. In addition to these workshops each room is asked to nominate at least one communication champion who commits to attending three Communication Champions workshops over the course of the academic year. These workshops focus on providing staff with the skills necessary to support the successful implementation of Happy Talk in their setting. Optional themes include multiculturalism; early literacy; engaging parents; and identifying speech, language and communication needs. Workshops are delivered once a term, and last 2.5 – 3 hours. The Happy Talk pre/school program is very well established and has been successfully implemented in 72 settings in one region in Ireland over the past 12 years. The feasibility and acceptability of the programme to parents and pre/school staff has therefore been established.

**Fidelity.** Each aspect of the Happy Talk programme is outlined in a manual which has been introduced as part of the SLT training to facilitate Happy Talk. Training included 1) how to coach parents and staff, 2) how to engage parents 3) how to work in disadvantaged communities and 4) how best to build social capital between therapists, parents, and early years educators, with the aim of increasing engagement with the intervention. The Happy Talk team administering the training will complete self-evaluation checklists for adherence to the core key elements and SLTs who attended the training will complete learning evaluation checklists.

To ensure SLT fidelity (for both parent and early educator components of the intervention) therapists will complete self-evaluation treatment fidelity checklists following each workshop and training and coaching sessions and adherence will be checked against the protocol laid out in the manual. All sessions will be audio recorded using a microphone worn by the interventionist, set at a level intended to capture their audio only. Each week two members of the research team will observe and review 20% of the recorded sessions and will independently complete the same fidelity checklists as those completed by the SLTs. In addition, dosage and accuracy with which clinicians deliver the elements specified in the manual, will be noted, and extrapolated up from the 20% viewed. Checklists completed by the SLTs will be compared with those completed by the research team and feedback will be provided. Overall fidelity will be calculated by summing scores for each of the individual components where there is agreement. Furthermore, the 8 SLT interventionists (4 each year) will be given ongoing support throughout the intervention through a once weekly video or audio call from the lead Happy Talk clinician.

***Comparator interventions***

Comparator interventions are the ‘business as usual’ Early Childhood Care and Education programme (ECCE)) and national Junior infant class curriculum. The ECCE programme is a national universal two-year pre-school programme available to all children between 2yrs 8 months and 5yrs 6 months. It provides children with their first formal experience of early learning prior to commencing primary school. The programme is provided for three hours per day, five days per week over 38 weeks per year and the programme year runs from September to June each year. Childcare services taking part in the ECCE programme must provide an appropriate pre-school educational programme which adheres to the principles of Síolta, the national framework for early years care and education. The most commonly implemented programme is ‘Aistear’ which is based on 12 principles of early learning and development, presented in three groups 1) children and their lives in early childhood 2) children’s connections with others and 3) how children learn and develop. *Communication and language* is one element of the third component.

Junior infants is the first year of an 8 year cycle in primary education. The primary curriculum is presented in 7 areas: Art; Mathematics, Social Environmental and Scientific Education; Physical Education; Religious Education; Primary Language; and Social, personal and Health Education. Any children who are receiving SLT in the community can continue to do so throughout the trial.

### Outcomes

***Primary- child***

The Pre-school Language Scales – 5 ^th^ Edition (PLS 5) ( Zimmerman *et al.*, 2014) is a standardized norm referenced language assessment that yields standard scores for total language, auditory comprehension, and expressive communication. A standard score of 100 represents the performance of a typical child at a given age. Standard scores between 85 and 115 correspond to one standard deviation below and above the mean, respectively; scores within this range are considered to be within normal limits.

The Focus on the Outcomes of Communication Under Six (FOCUS-34) ( Oddson *et al.*, 2019; Thomas-Stonell *et al.*, 2010) is a clinical tool designed to evaluate change in communicative-participation in preschool children. We have obtained a copyright license to use this tool. The parent form consists of 34 statements, aimed at taking a snapshot of children’s skills as they are on that day. Example statements include, *my child can tell stories that make sense; my child will ask for things from other children*. Parents are asked to rate each statement using a 7 point scale, ranging from ‘not at all like my child’ to ‘exactly like my child’. This yields a total score ranging from 50 to 350 with a higher score indicating a better outcome.

***Secondary - child***

PedsQL ^TM^ ( Buck, 2012) is a standardized, parent proxy-report scale of health-related QoL in young children. The PedsQL contains 23 items and measures four health dimensions: physical, emotional, social, and school functioning (questions related to school or daycare if attended). The tool asks, “please tell us how much of a problem each item has been for your child during the past one month.” Parents are required to rate each item on a scale of 0–4 (0 indicating never a problem and 4 almost always a problem). The ratings are tallied yielding a total score for each section, the higher score indicating a greater level of difficulty.

The Child Health Utility instrument (CHU9D) ( Furber & Segal, 2015), (caregiver completed) is a generic preference- based measure, using parent report scales to measure health related quality of life, in young children. The CHU9D (for children < 5years) consists of 11 questions and parents are asked to base their responses on how their child is feeling on the day of completion. It consists of a descriptive system and a set of preference weights, which give utility values for each health state described by the descriptive system, allowing the calculation of QALYs ( Mpundu-Kaambwa *et al.*, 2017). 

***Secondary - parent***

The Maternal Responsive Behaviours Coding Scheme (MRBCS - Levickis *et al.*, 2014) is an observational coding scheme of parent– child interaction. Implementation of the MRBCS yields a total number of occurrences for each of the four parental responsive behaviours (Expansions; Imitations; Responsive Questions; and Labels), for a given period. By summing the frequency scores for each behaviour, an overall score of parental responsiveness can be calculated. The higher the score the greater the number of parental responsive behaviours - yielding better outcomes.

***Secondary - setting***

The Classroom Assessment Scoring System (CLASS; La Paro & Pianta, 2003) is designed to assess the quality of interactions between teachers and students in the classroom. The CLASS measures three broad domains of teacher-student interactions (Emotional Support, Classroom Organization, and Instructional Support) which are assessed across 10 specific dimensions: positive climate, negative climate, teacher sensitivity, regard for student perspectives, behavior management, productivity, instructional learning formats, concept development, quality of feedback, and language modeling. Assessors observe classrooms for a minimum period of 2 hours and assign scores on each dimension using 7-point scale.

### Recruitment

A short recruitment video will be made to explain the purpose of the study and what it entails. The clinical project manager, will meet with the local childcare committee manager; preschool managers; and school principals in each potential area, to answer any questions about the programme. Parents and early years educators will also be given information leaflets and two members of our PPI group will be involved in talking to parents and staff about the study. All preschool children enrolled in the National Childcare scheme (aged between 2 years and 8 months and 5 years and 6 months) or attending Junior Infants in the educational settings recruited for the project, their parents/caregivers, and a minimum of 1 educator at each setting, will be invited to participate in the project. It will be made explicit from the outset that all settings will not receive the intervention. Recruitment will take place over a period of two months (for each iteration). Pre/school managers/principals interested in having their educational setting participate in the project will provide verbal consent over the phone. Subsequently, they will receive a consent form via email, which will be collected by the team member responsible for conducting the research activities in the pre/school. Parents/caregivers that wish to participate in the project will be requested to return a signed consent form to the principal/manager of their school/preschool. Child participants will be recruited via their parents/caregivers.

### Data collection

Baseline assessments (all outcome measures) will be completed for children, parents, and each pre/school setting before being randomised to the experimental or control condition. Outcome measures will also be administered immediately post intervention, and at 6 months follow up. In addition, quality of life measures will also be administered at 1 year follow up. Outcome measures will be administered by the two research assistants employed on the project and 12 additional research assistants employed for the assessment periods only (all of whom will have some experience administering standardized assessments). Demographic information will be gathered from families to establish socio-economic status at the level of the child (measured through education of the primary caregiver) and if there is any history of speech and language difficulties in the family. Background information about the children will also be gathered including any diagnosed hearing difficulties, exposure to additional languages or whether they are already attending SLT. All parents taking part in the evaluation will receive a 20 euro ‘child-focused’ voucher at each assessment time point. All settings will receive a series of children’s books and control settings will receive continued professional development workshops post-intervention. Parental and educators’ intervention attendance levels will also be documented.

***Training***

Training will be given in the administration and coding of each measure as follows.

- Assessors administering the *PLS-5* will attend a training day with a senior SLT who has experience using this assessment.
- Assessors administering the FOCUS and the quality of life measures with parents, will be familiarised with each measure and attend a question/answer session about administration.
- Those administering *The Maternal Responsive Behaviours Coding Scheme* will complete a training protocol under the guidance of the author of the coding scheme, where they will rate videos which will be compared with the ratings of the author. A criterion of 80% agreement will be required before progressing to rate the study videos.
- Research assistants administering or involved in reliability checking of the Classroom Assessment Scoring System (CLASS) will attend a two-day Observation Training provided by a certified CLASS trainer. Assessors will be required to code video segments online. To pass reliability they will be required to score within 1 point of the master code on 80% of all codes given, across all ten CLASS dimensions. The observation training will be given by the chief investigator who will complete the CLASS train-the-trainer programme online.

***Data coding and reliability***

Primary Outcome Measures:

- PLS-5: Children’s responses will be scored live according to the instruction manual, while administering the assessment. A second research assistant blinded to the participants’ group allocation, will independently examine 20% of randomly selected assessments to determine the reliability of the online scoring (checking total raw score accuracy and conversion to standard scores). Point-to-point agreement will be calculated between scorers.
- FOCUS ©- Will be completed over the phone with parents. The research assistants will document parent responses. 20% of the forms will be randomly selected for rescoring.

Secondary Outcome Measures:

- Child: QoL measures will be administered by phone and scored ‘live’ by the RAs during each phone interview. 20% of the forms will be randomly selected for rescoring.
- Parent: The MRBCS videos will be rated by an RA trained in the use of this measure, who is blind to time and group. 20% of the videos will be randomly selected for double coding by another RA, again blind to time and group.
- Pre/school: Trained testers will administer the CLASS independently ‘live’ in each pre/school (designated as primary observers). 20% of the settings will be randomly selected to be rated by an additional observer and reliability will be reported.

### Data management

Quantitative variable measurements will be recorded in international standardized units. Variables will be recorded within Comma Separated Value (.csv) data tables and range checks will be conducted for data values. Data files will include meta-data identifying title, creator, keywords etc. in line with the Dublin Core Metadata Initiative. Accompanying data dictionaries will be developed in parallel. The Electronic Data Capture platform (Castor EDC) will be used throughout this project. The Castor EDC platform contains in-built audit trail supports and data validation processes aligned to enact FAIR data outputs. Qualitative data will be imported into an NVivo database to facilitate analysis. Data will be encrypted at rest and in transit, two-factor authentication at log in, and daily backup and file syncing. All data will be stored so that it is amenable to audit. The PI (Professor Pauline Frizelle) is responsible for data governance and the research team are working closely with the university data steward to create a comprehensive data management plan.

RAs will digitise parents’ responses into Castor at collection

### Statistical considerations

***Planned sample size***

The trial is designed to have reasonable power to detect a modest intervention effect. Using *a priori* power calculations based on the goal of detecting an effect size of Cohen’s d = 0.25, with a power of 80%, an intra class correlation of .05 (with an average of 10 participants per cluster) and a standard significance level of 5%, the required number of clusters is 72. We targeted a modest effect size based partly on the 1) the pilot trial of Happy Talk ( Frizelle *et al.*, 2021b), which showed standardized effect sizes of 0.6 SD ( *p* = 0.005), 0.21 SD ( *p* = 0.26) and 0.46 SD ( *p* = 0.01) for receptive, expressive and total language scores, respectively; 2) the empirical literature which shows an effect of d = 0.3 for expressive language ( Heidlage *et al.*, 2020); and 3) research showing that intervention effects are often reduced when implemented at scale ( Bleses *et al.*, 2021). Further, the sample size calculations are conservative in that they do not account for efficacy gains following from stratified randomization; subsequent statistical adjustment for said stratifiers; and additional, prognostic participant- and cluster-level covariates in analyses of the trial data. Finally, we will include an additional 2 participants per cluster to allow for loss to follow-up, based on retention figures from our pilot trial, resulting in a target of 864 total participants enrolled in the trial.

***Randomisation, allocation concealment and blinding***

At the start of each of the two study cycles, 36 of 72 settings will be randomly allocated to either the Happy Talk or the “business as usual” study arms following their enrolment. The randomization will be stratified by geographical region and preschool versus school and restricted within strata using a block size of 2. Then, from the consenting families within each setting, 12 children and their parents/ caregivers will be randomly chosen at the start of the school year for assessment of study outcomes. The randomization lists for both settings and children/families will be prepared under the Standard Operating Procedures of the HRB-CRF-UCC. Allocation concealment will be maintained through the use of a protected electronic database so that the allocation will only become available to interventionists, when pre/schools and their associated families have unambiguously consented and enrolled onto the trial and when baseline measures have been completed. The study will then be open label from that point, as both participants and study staff delivering the interventions will be aware of how settings were allocated. However, outcome assessors will remain blind to allocation, and the initial analyses of the study data will be done by a blinded study statistician.

***Data analysis***

Analyses of study outcomes will be done at the participant level, and not based on cluster level summaries of participant data. Further, while we don’t expect cross-over between study arms, all participants will be analysed as members of the arm they were randomized to. Further, given the pragmatic focus on the trial, participants’ data will be analysed without consideration for their degree of engagement, or lack thereof, with the study interventions (using an intention to treat analysis). Supplementary analysis will examine the impact of high versus low engagement across parents and educational settings.

Given the Happy Talk design, between arm differences in the primary child language outcomes (PLS-5, FOCUS 34) measured immediately post-intervention and 6 months later will be estimated using linear mixed effects models for repeat measures ( Bell & Rabe, 2020) with a random effect for setting, and fixed effects for study arm, timepoint, baseline outcome scores, study cycle, and geographic region (following from the stratified randomization). We will also use a second set of similar models that further adjust for setting type and gender mix, and child age, primary caregiver education, family history of speech and language difficulties, diagnosed hearing difficulties, exposure to additional languages, and any current or previous SLT. Our estimates of between arm differences in timepoint specific outcomes will be reported alongside 95% frequentist confidence intervals without consideration for multiple comparisons ( Althouse, 2016). Missing data will be carefully evaluated and a plan for addressing them will be based on what we observe. However, our planned mixed effects models for repeat measures will produce unbiased estimates assuming data are missing at random ( Bell & Rabe, 2020), conditional on model covariates. Analyses for secondary outcomes will follow a similar strategy using generalized mixed effects models for repeat measurements, using a link function appropriate for each outcome.

All analyses will be conducted under the quality system of the HRB CRF-UCC. A statistical analysis plan is available on the Open Science Framework ( https://osf.io/ns7u5/). Any necessary deviations from this SAP will be documented and explained in the trial report. All reporting will carried out in accordance with CONSORT guidelines for clinical trials.

***Data monitoring***

As this is a low risk trial (of a complex intervention), the Steering Committee will also act as the Independent Data Monitoring Committee (IDMC). They will monitor the safety of participants and make recommendations to the management group in relation to ethical issues and maintaining the integrity of the trial. To ensure protocols in relation to governance and safety monitoring are strictly adhered to, the steering committee includes personnel who can monitor and give expert advice that is completely independent of the PI; the trial managers; the research team members; and the institutions involved. More details regarding steering committee membership are provided in the supplemental material.

Because data collection will take place over two cycles, we will have an opportunity to conduct an interim analysis at the end of the data collection completed for the first cycle. At this stage, we will know if the cycle 1 recruitment targets were met, and also be able to estimate the degree of clustering and variance we observed for the outcomes. This information will in turn facilitate a sample size re-evaluation that can inform how the study proceeds for the second cycle. If the re-analysis suggests that an increased sample size target is required for cycle two, this will be carefully considered by the Trial Steering Committee (if no such increase is suggested, then the study will proceed as planned). At this stage, the Trial Steering Committee will have the option of requesting a formal futility analysis to help determine whether the trial should continue or be stopped prior to data collection in year two. Details of the analysis and the results of the Trial Steering Committees’ deliberations will be reported back to the funder and a final decision will be negotiated by all stakeholders.

### Economic evaluation

Although the economic benefits of investing in early intervention for children are well established ( Heckman, 2006), and cost effectiveness has been examined in relation to education and health programmes with broad outcomes (e.g. Knight *et al.*, 2019; Ludwig & Phillips, 2008), limited work has been done modelling the immediate or long term economic benefits of speech and language interventions ( Le *et al.*, 2020).

An economic evaluation will compare the costs and effects of the Happy Talk intervention, to usual care. The evaluation will also include a budget impact analysis, which predicts the potential financial impact of the adoption and diffusion of Happy Talk, to inform resource or budget planning. The evaluation builds on a previous economic evaluation of our small-scale effectiveness trial ( Frizelle *et al.*, 2021a). The preliminary economic evaluation included a value of information analysis that suggested there was value in collecting further information. This scaled up, full definitive trial will a) provide updated parameter estimates on costs and effectiveness; b) reduce uncertainties around parameter estimates and c) reduce uncertainty around cost effectiveness estimates.

Following standard Health Information and Quality Authority (HIQA) guidelines on conducting economic evaluations a cost utility analysis will be undertaken. In the baseline analysis the perspective of the service provider will be adopted, thus only direct resources utilised will be included. All resources utilised for the delivery of the intervention and standard care will be identified, measured and valued using micro-costing techniques. Resources utilised will be captured by a dedicated resource utilisation questionnaire.

The evaluation will include a primary, secondary and sensitivity analyses. In our primary cost utility analysis effectiveness will be measured using standardised Health Related Quality of Life (HRQoL) questionnaires pre-, post- and at 12 months follow-up. HRQoL measures enable the calculation of Quality Adjusted Life Years (QALYs) as well as an investigation of the sensitivity of these measures with children. To date the use of child appropriate HRQOL is limited, with many studies employing measures and health state values intended for adults. In addition, no health technology assessment (HTA) agency worldwide provides methods guidance on measuring HRQoL in young people. Given persistent issues with valuing child HRQoL, including expected delayed differences in HRQoL and lack of value sets, clinical measures (The PLS-5 and the Focus-34) will be used to test the robustness of results in our secondary analysis. Lastly, the sensitivity analysis will assesses robustness of the effectiveness measures, uncertainty in input and resulting output parameters.

*Analysis:* Difference in costs and effects between the intervention and standard care will be estimated and an Incremental Cost Effectiveness Ratio (ICER) will be estimated. For the baseline analysis if this ICER is less than the nationally accepted threshold (€45,000/QALY) the intervention could be considered cost effective compared to standard care. Results of the probabilistic sensitivity analysis will be presented on Incremental Cost Effectiveness Planes and Cost Effectiveness Acceptability Curves to investigate uncertainty surrounding the output parameters and cost effectiveness decision. The cost results in the economic evaluation will inform the Budget Impact Analysis which will examine budgetary implications associated with national scaling up the intervention over a 5-year period (as per HIQA guidelines).

## Discussion

The Happy Talk trial aims to mitigate the effects of social disadvantage on child language outcomes and therefore improve the overall life chances of children living in deprivation. The programme is already well established in one region of Ireland and has been shown to be both feasible and acceptable to parents, early years educators and speech and language therapists. The trial is a large scale ‘real world’ effectiveness trial, embedded in the community, which engages with parents and educators, and as such responds to identified evidence gaps. The trial includes an at scale economic evaluation from the perspective of the healthcare provider and outcomes address both functional and standardized measures.

If effective we anticipate the following outcomes:

- Evidence that receptive and expressive language difficulties and their functional manifestation can be improved in pre- and early school years, in a cost effective manner, using a public health service delivery model.
- The availability of a well evaluated speech, language and communication intervention which can be implemented by speech and language therapists in the community,

## Ethics and consent

The study has been reviewed and approved by the Reference Research Ethics committee, Health Services Executive, Dublin and Midlands (with remit for National approval) – RRECB0124PF (14 ^th^ March, 2024). Any changes to the protocol will be submitted as an amendment to the original submission. To obtain informed consent, each SLT, educational setting (preschool manager/school principal), educator, parent/ caregiver and child will be provided with an information letter and consent/assent form, adapted to their specific group.

Participation in the study and the identity of the participants will be treated as confidential and no participant identifiable records or results relating to the study will be disclosed to any third party other than the authorized investigators. However, if children are identified as having significant speech and language difficulties that require individual intervention, families will be supported by their interventionist for onward referral.

Personal identifiers will undergo pseudonymization. An encryption key, held securely away from the data, will be accessible to the project PI at site only. In line with the funders (Health Research Board) policy for post-project data publication, consent will be obtained at recruitment for all anonymized data to be shared publicly on an open science repository. Careful evaluation and assessment of publishable data catalogues will be continually reviewed to ensure that data objects will be ‘as open as possible, yet as closed as necessary’ upon completion of the research. The use of data repositories will ensure maximum utility and interoperability of the final data package(s) and assignment of a persistent digital object identifier (DOI). Additional post-study data provenance will be enacted through sharing of analysis scripts and study protocols via the Open Science Framework and/or the HRB Open Research platform projects with accompanying DOI(s). Study findings will be written up as journal publications, and published open access. Findings will also be disseminated to our participants through lay summaries on the project website and will presented to the CHO policy makers at a national meeting. Project co-applicants and collaborators will be eligible for authorship.

## Data Availability

No new data are associated with this article. Extended data: Extended data are available on the Open Science Framework. *Evaluating a targeted selective speech, language, and communication intervention at scale – the Happy Talk cluster randomised controlled trial* (DOI 10.17605/OSF.IO/NS7U5) ( Frizelle *et al.*, 2024) The project contains the following underlying data. Child Participant Trial and Process Evaluation information leaflet Parent Caregiver Participant Trial and Process Evaluation Consent form Parent Caregiver Participant Trial and Process Evaluation information leaflet Educator Participant Trial and Process Evaluation information leaflet SLT and Educator Participant Trial and Process Evaluation consent form Resource Utilisation Questionnaire Statistical Analysis plan SPIRIT Checklist Trial Governance WHO Trial Registration Dataset Data are available under a CCO license.
