# Supplementary material for: Evaluating a targeted selective speech, language, and communication intervention at scale – Protocol for the Happy Talk cluster randomised controlled trial
Source: HRB Open Res. 2025 Jan 31;7:65. Originally published 2024 Oct 8. [Version 3] doi: 10.12688/hrbopenres.13973.3 (PMC11808849; doi:10.12688/hrbopenres.13973.3)
Supplement: Peer review history

# Reviewer report: Reviewer response for version 3

**DOI:** 10.21956/hrbopenres.15461.r45226

**Published:** 2025-02-04

**Authors:** Naja Ferjan Ramirez, Adeline Braverman

**Affiliations:** 1 University of Washington, Seattle, USA; 2 University of Washington Seattle, USA, USA; 3 Linguistics, University of Washington (Ringgold ID: 7284), Seattle, Washington, USA

I think the authors have adequately addressed our concerns/questions.

Is the study design appropriate for the research question?

Partly

Is the rationale for, and objectives of, the study clearly described?

Yes

Are sufficient details of the methods provided to allow replication by others?

Partly

Are the datasets clearly presented in a useable and accessible format?

Not applicable

Reviewer Expertise:

Language acquisition in infancy and early childhood; Language intervention; Language input

We confirm that we have read this submission and believe that we have an appropriate level of expertise to confirm that it is of an acceptable scientific standard.

# Reviewer report: Reviewer response for version 3

**DOI:** 10.21956/hrbopenres.15461.r45223

**Published:** 2025-02-04

**Authors:** Nicola Botting

**Affiliations:** 1 City University London, London, UK

Thank you for detailed responses to my comments. I am happy to approve version 3.

Is the study design appropriate for the research question?

Yes

Is the rationale for, and objectives of, the study clearly described?

Yes

Are sufficient details of the methods provided to allow replication by others?

Yes

Are the datasets clearly presented in a useable and accessible format?

Not applicable

Reviewer Expertise:

Developmental Language Disorder & other forms of atypical language development

I confirm that I have read this submission and believe that I have an appropriate level of expertise to confirm that it is of an acceptable scientific standard.

# Reviewer report: Reviewer response for version 2

**DOI:** 10.21956/hrbopenres.15422.r44222

**Published:** 2025-01-11

**Authors:** Naja Ferjan Ramirez, Adeline Braverman

**Affiliations:** 1 University of Washington, Seattle, USA; 2 University of Washington Seattle, USA, USA; 3 Linguistics, University of Washington (Ringgold ID: 7284), Seattle, Washington, USA

This is a revised version of the narrative, and I think most of the issues have already been addressed. Overall, the study is well-designed and targets an important public health concern. We are pointing out some potential areas of improvement.

 12 1-hr sessions attended by parents: This could be a challenge as it is quite intensive and requires a lot of time and effort. Parents are busy, and may not be able to attend all (or even most) sessions – this seems especially relevant given that families are low SES. How will the team make sure that parents attend the sessions? The narrative suggests that this intervention has already been implemented successfully in parts of Ireland. Is there any data to show that parents stayed engaged throughout the program? On a related note, were parents invited to help in the DESIGN of the intervention? Do we know that they find it (culturally) acceptable and doable/executable given their day-to-day lives? Not much is said about this in the narrative.

 The second research question asks whether Happy Talk “enhance[s] responsiveness and language-promoting behaviors in home and pre/school contexts.” The associated hypothesis is that intervention parents will have higher Maternal Responsive Behaviors Coding Scheme (MRBCS) scores. Is the MRBCS sensitive to “language-promoting behaviors” beyond those associated with responsiveness? The research question may need to be narrowed so that it is adequately addressed by this outcome measure, or the authors should clarify that responsiveness is the language-promoting behavior in question.

 Nothing is said about measuring “attendance” – i.e. are the effects of the intervention hypothesized to be weaker in those families who attend fewer sessions? Relatedly, what were the attendance/drop-out rates in previous iterations of the intervention?

 Inclusion of fathers: not much is said about this…in our experience, if fathers are just “invited”, they tend to not attend the sessions. Will there be an explicit effort to recruit and retain the fathers? Why (not)? OR – alternatively -  if both parents attend in some cases, and only one parent attend in others, can there be supplemental hypotheses around whether one or both parents received the treatment?

 In the ‘Data Collection’ section, it is somewhat unclear what measures will be collected at the Baseline Assessment. Are all outcome measures included at this initial timepoint?

 How will the PLS-5 be scored by the second research assistant? Will these assessments be recorded?

 Preschool staff treatment – 4x 2.5-3 hours of staff training: similarly to parent training, do we know that the staff are eager and excited to undergo these trainings? What about the school administration? Will they be able to pull the teachers out of the classroom to give them the necessary time for training? On a related note, are teachers engaged in the design of the intervention? If so, what are their opinions of it? If not, they may find it challenging to implement.

 Child outcomes: most are parent/staff reports, which is a disadvantage, given that they’re being treated. One might hypothesize that the intervention will train the parents/staff to answer the questionnaires differently compared to the control group. I recommend that the team add child outcome language measures that are NOT questionnaire based. This was already noted by a previous reviewer. In their response, the authors cite work demonstrating the reliability and validity of parent report instruments, but this work focuses only on the MBCDI in a younger age range than the proposed sample and outside the context of parent-mediated intervention.

 In Figure 2, the use of multiple arrows leads the reader to believe that each bullet point is associated with another in the neighboring box. However, the bullet points do not always line up (in number and content). Consider reducing the number of arrows or clarifying the connections between the contents of each box.

 For the most part, there is sufficient detail of the methods provided to allow for replication. The hypotheses and the rationale, the subject numbers and characteristics are well described and make sense. The only thing that’s missing is the intervention “scripts”/ content/ manual – this is not shared, so one could not actually replicate the study as it is unclear what the parents / staff are actually told during the sessions. I am not sure if this is intentional.

Is the study design appropriate for the research question?

Partly

Is the rationale for, and objectives of, the study clearly described?

Yes

Are sufficient details of the methods provided to allow replication by others?

Partly

Are the datasets clearly presented in a useable and accessible format?

Not applicable

Reviewer Expertise:

Language acquisition in infancy and early childhood; Language intervention; Language input

We confirm that we have read this submission and believe that we have an appropriate level of expertise to confirm that it is of an acceptable scientific standard, however we have significant reservations, as outlined above.

# Response

**Published:** 2025-01-23

**Authors:** Pauline Frizelle

**Affiliations:** Speech and Hearing Sciences, University College Cork, Cork, Ireland

Many thanks for your valuable feedback, we have addressed each point in turn.

**12 1-hr sessions attended by parents: This could be a challenge as it is quite intensive and requires a lot of time and effort. Parents are busy, and may not be able to attend all (or even most) sessions – this seems especially relevant given that families are low SES. How will the team make sure that parents attend the sessions? The narrative suggests that this intervention has already been implemented successfully in parts of Ireland. Is there any data to show that parents stayed engaged throughout the program? On a related note, were parents invited to help in the DESIGN of the intervention? Do we know that they find it (culturally) acceptable and doable/executable given their day-to-day lives? Not much is said about this in the narrative.**

**Response: **Happy Talk has been delivered in one part of Ireland for 12 years. The programme was originally developed by SLTs but the design has been adapted over the years based on feedback from parents and early years educators.  We also measured feasibility and acceptability in a new area in our pilot trial (referenced within). As stated in response to reviewer 2, in areas where Happy Talk is well established there is very high attendance as other parents/educators have spoken so positively about the programme and parents have an expectation that this will be part of their child’s education. In new areas (as is the case here) attendance tends to increase as the programme progresses. Parents become more committed as their confidence increases, and children request their parents to be present in the classroom, as they enjoy this aspect of the programme. In any case, we take a conservative approach in using an intention to treat protocol, so that if a parent attends even one session they are considered to have received the intervention.

**The second research question asks whether Happy Talk “enhance[s] responsiveness and language-promoting behaviors in home and pre/school contexts.” The associated hypothesis is that intervention parents will have higher Maternal Responsive Behaviors Coding Scheme (MRBCS) scores. Is the MRBCS sensitive to “language-promoting behaviors” beyond those associated with responsiveness? The research question may need to be narrowed so that it is adequately addressed by this outcome measure, or the authors should clarify that responsiveness is the language-promoting behavior in question.**

**Response:** We have clarified that responsiveness is the language-promoting behavior in question.

**Nothing is said about measuring “attendance” – i.e. are the effects of the intervention hypothesized to be weaker in those families who attend fewer sessions? Relatedly, what were the attendance/drop-out rates in previous iterations of the intervention?**

**Response:** We have clarified that attendance will be measured throughout both iterations of the programme. As stated above, attendance tends to increase as parents become more engaged with the programme. We have reported the attendance rates and attrition in our published pilot trial.

**Inclusion of fathers: not much is said about this…in our experience, if fathers are just “invited”, they tend to not attend the sessions. Will there be an explicit effort to recruit and retain the fathers? Why (not)? OR – alternatively -  if both parents attend in some cases, and only one parent attend in others, can there be supplemental hypotheses around whether one or both parents received the treatment?**

**Response:** Given the demands of the programme the reviewer has highlighted, we encourage one parent/caregiver to attend, whichever places the smallest burden on the family. In our experience this is most often the mother but fathers and grandparents have also attended. If there are a sufficient number of cases where individual parents attend different sessions or both parents attend we will examine any potential effects statistically. However, this has not been our experience in the past. We request that the same person attend for baseline and end of programme measures.

**In the ‘Data Collection’ section, it is somewhat unclear what measures will be collected at the Baseline Assessment. Are all outcome measures included at this initial timepoint?**

**Response**: We have clarified that all outcome measures are included at the initial stages.

**How will the PLS-5 be scored by the second research assistant? Will these assessments be recorded?**

**Response:** No, these are not recorded. The 2 ^nd^ researcher will check that the scores have been added correctly, and converted accurately into standard scores. We have clarified this in the text.

**Preschool staff treatment – 4x 2.5-3 hours of staff training: similarly to parent training, do we know that the staff are eager and excited to undergo these trainings? What about the school administration? Will they be able to pull the teachers out of the classroom to give them the necessary time for training? On a related note, are teachers engaged in the design of the intervention? If so, what are their opinions of it? If not, they may find it challenging to implement.**

**Response**: In keeping with my response with respect to parents, teachers have implemented the programme in one area of Ireland for many years, and have reported it to be feasible and acceptable – we have noted this in the text. They have also had input into the programme as it was developing over many years. We carry out the sessions in the classroom at a time that is convenient for the teachers. In addition, all teachers are required to engage with continuous professional development, the Happy Talk training fulfils that need.

**Child outcomes: most are parent/staff reports, which is a disadvantage, given that they’re being treated. One might hypothesize that the intervention will train the parents/staff to answer the questionnaires differently compared to the control group. I recommend that the team add child outcome language measures that are NOT questionnaire based. This was already noted by a previous reviewer. In their response, the authors cite work demonstrating the reliability and validity of parent report instruments, but this work focuses only on the MBCDI in a younger age range than the proposed sample and outside the context of parent-mediated intervention.**

**Response**: We have included the PLS-5 which is a standardized language measure which provides a receptive, expressive and composite language score.

**In Figure 2, the use of multiple arrows leads the reader to believe that each bullet point is associated with another in the neighboring box. However, the bullet points do not always line up (in number and content). Consider reducing the number of arrows or clarifying the connections between the contents of each box.**

**Response: **Where arrows don’t directly align they are intended to refer to more than one component within a given box, we have clarified this in the text.

**For the most part, there is sufficient detail of the methods provided to allow for replication. The hypotheses and the rationale, the subject numbers and characteristics are well described and make sense. The only thing that’s missing is the intervention “scripts”/ content/ manual – this is not shared, so one could not actually replicate the study as it is unclear what the parents / staff are actually told during the sessions. I am not sure if this is intentional.**

**Response:** We cannot make the manual publicly available due to copyright reasons. Those who are interested in getting further information, or adapting the intervention to their context are welcome to get in touch with the first author.

# Reviewer report: Reviewer response for version 2

**DOI:** 10.21956/hrbopenres.15422.r43956

**Published:** 2025-01-08

**Authors:** Nicola Botting

**Affiliations:** 1 City University London, London, UK

This is a very well constructed and clear protocol for a large scale intervention trial using the Happy Talk programme. There is substantial need for the evaluation of early years language interventions such as this, and this team is ideally placed to carry out such research.  The previous 2 reviewers have given comprehensive and insightful comments, many of which have already been responded to by the authors, therefore my observations are brief and minor.  Some of them echo other points already made.

 1. RQ1: It might be helpful for readers to include 'compared to usual care' in RQ1. This is implied, but at the moment leaves the possibility that positive within-group change for the intervention group alone could answer this question (which is not the authors' intention).

 2. Recruitment: Opt-in consent is being used.  It would be useful to identify and detail any strategies for monitoring/ensuring that these sign ups represent low SES groups, as even within schools in disadvantaged areas, participation from some groups is difficult to attain.  Would opt-out consent be considered if recruitment shows bias? Have the authors considered adding the option for a Qualtrics (or similar) link to consent, which we are finding useful in recruiting families where bits of paper get lost (I acknowledge this method has its own inherent biases, but maybe a combination?). Under Randomisation, it could be made clearer that the families are randomly selected *from consenting families*. The inclusion of families with additional languages is important, but it is not clear to me how the parent English level will be assessed.  A small point but it is probably best to avoid the term 'subjects' even in a trial?

 3. Data management:  I have no doubt that this team are thoroughly conversant in data management, but for readers and replicability more detail about how data sharing (agreements), and transit across sites (and countries) will be securely achieved would be welcome.  It would be useful to know who has overall responsibility for data governance and data backups etc.

 4. Training workshops: It was not clear to me how many people are at each workshop or included in the weekly video-conference support sessions. This detail seems important in terms of attendance bias, and success of the programme, and is needed for replicability. It would be useful to specify whether by 'recorded' you mean video or audio only.  How will you deal with participants who do not give consent to recording or to other people in the background?

 5. Assessment measures: In line with the other reviews, I am not sure about the CLASS and wondered whether authors had considered the CsC developed by Dockrell et al. for Better Communication?  Will the reliability measures taken by an additional person occur at the same time or on a different day?  In addition I wondered whether the FOCUS-34 has a reference?  A couple of example questions might be useful here.  For the MRBCS, I think this should read 'a total number of occurrences for each of four parental behaviours'?  If not, more explanation is needed.

 6. Economic assessment: I agree with other reviewers that it is confusing to use only the HRQoL measure for this stage of the project, especially when this didn't change on pilot trials. I see in the author response that the PLS-5 and FOCUS-34 are now included in a secondary economic analysis, but cannot find this detail in the protocol itself. 

 Overall, this is a comprehensive and impressive protocol which has potential to make substantial impact and contribution. The minor suggestions above are mainly intended to enhance replicability and to increase usefulness even further.

Is the study design appropriate for the research question?

Yes

Is the rationale for, and objectives of, the study clearly described?

Yes

Are sufficient details of the methods provided to allow replication by others?

Yes

Are the datasets clearly presented in a useable and accessible format?

Not applicable

Reviewer Expertise:

Developmental Language Disorder & other forms of atypical language development

I confirm that I have read this submission and believe that I have an appropriate level of expertise to confirm that it is of an acceptable scientific standard.

# Response

**Published:** 2025-01-23

**Authors:** Pauline Frizelle

**Affiliations:** Speech and Hearing Sciences, University College Cork, Cork, Ireland

Thank you for taking the time to give your valuable feedback.

 1. RQ1: It might be helpful for readers to include 'compared to usual care' in RQ1. This is implied, but at the moment leaves the possibility that positive within-group change for the intervention group alone could answer this question (which is not the authors' intention).

**Response**: We have added the clarification to RQ1 - that the intervention is being compared to usual care

 2. Recruitment: Opt-in consent is being used.  It would be useful to identify and detail any strategies for monitoring/ensuring that these sign ups represent low SES groups, as even within schools in disadvantaged areas, participation from some groups is difficult to attain.  Would opt-out consent be considered if recruitment shows bias? Have the authors considered adding the option for a Qualtrics (or similar) link to consent, which we are finding useful in recruiting families where bits of paper get lost (I acknowledge this method has its own inherent biases, but maybe a combination?). Under Randomisation, it could be made clearer that the families are randomly selected *from consenting families*. The inclusion of families with additional languages is important, but it is not clear to me how the parent English level will be assessed.  A small point but it is probably best to avoid the term 'subjects' even in a trial?

**Response:** In relation to the profile of those who sign up, we appreciate that there will be variability in terms of levels of disadvantage. However, we will note these individual differences in the collection of our demographic data. As stated in response to another reviewer  we acknowledge that the same variation will exist within the control settings and therefore should not be a confounding variable.

 In addition to the initial information session, our consent process is being facilitated by the pre/school staff who are available to read through the trial information and consent forms and answer any questions that potential participants might have when dropping and collecting their children. We appreciate the suggestion re Qualtrics but this would require access to a computer and feedback from pre/school staff is that they prefer to support the process in an informal way so that parents don’t feel under pressure to participate in the evaluation.

 There are large numbers of children receiving the intervention who are not part of the evaluation and if we were to engage in an opt out process, we would not have the capacity to assess them all in the restricted time frame.

 We have clarified that families are randomly selected from consenting families.

 As the reviewer rightly points out we are not actively assessing parents level of English and we have amended the text to remove the requirement of level B2.  Parents will be asked if they are confident to complete the outcome measures without the need for an interpreter.

 We have replaced the term subjects, the use of which was an oversight on our part.

 3. **Data management:**  I have no doubt that this team are thoroughly conversant in data management, but for readers and replicability more detail about how data sharing (agreements), and transit across sites (and countries) will be securely achieved would be welcome.  It would be useful to know who has overall responsibility for data governance and data backups etc.

**Response:** This is a national study and therefore there is no data sharing between countries. We have made our data management plan available on OSF ( https://osf.io/ns7u5/) to address the reviewers point.

 4. Training workshops: It was not clear to me how many people are at each workshop or included in the weekly video-conference support sessions. This detail seems important in terms of attendance bias, and success of the programme, and is needed for replicability. It would be useful to specify whether by 'recorded' you mean video or audio only.  How will you deal with participants who do not give consent to recording or to other people in the background?

**Response: **The workshops are for the pre/school staff and will be attended by the number who are participating in the intervention in a given pre/school, this varies from setting to setting and goes beyond those who are included in the trial. For example in some preschools there may be 3 staff (working in one room) where as others may have 5 across two rooms. ****We have clarified this in the text.

 We have specified that we will audio record the sessions, the therapist will wear a microphone, set to capture his/her input only. We appreciate that some child audio may be picked up in the distance but they will not be identifiable; we will not transcribe it (if audible); and we will not be using it in any way to measure interventionist fidelity. We have clarified that there will be 4 SLTs each year (8 in total) who will be attending the video-conference calls. 

 5. Assessment measures: In line with the other reviews, I am not sure about the CLASS and wondered whether authors had considered the CsC developed by Dockrell et al. for Better Communication?  Will the reliability measures taken by an additional person occur at the same time or on a different day?  In addition I wondered whether the FOCUS-34 has a reference?  A couple of example questions might be useful here.  For the MRBCS, I think this should read 'a total number of occurrences for each of four parental behaviours'?  If not, more explanation is needed.

**Response:** We used the CsC in our pilot trial and although we were not able to complete statistical analyses (due to a small number of settings) our observations of the data suggested that it was not sensitive to the expected changes. Empirical literature suggests that the CLASS is a reliable tool across a number of languages (Hamre et al., 2008; Stuck et al., 2016; Virtanen et al., 2017) and we expect sensitivity to a number of areas in which we aim to effect change - in particular *Concept development, Quality of feedback *and *Language modelling*. In addition, colleagues who have significant experience researching in the area of professional development of early years educators, have found the CLASS to be a sensitive measure (Eadie et. al.,). Because the CLASS is completed live, reliability checking will involve score checking only. However, to be deemed a reliable coder, all CLASS administrators are required to score within 1 point of master codes on 80% of all codes given in the training videos.  

 We have added some references for the FOCUS-34, as well as some sample questions. We have reworded in relation to the MRBCS.

 6. Economic assessment: I agree with other reviewers that it is confusing to use only the HRQoL measure for this stage of the project, especially when this didn't change on pilot trials. I see in the author response that the PLS-5 and FOCUS-34 are now included in a secondary economic analysis, but cannot find this detail in the protocol itself.

**Response: **We have adapted the text to make the secondary (economic) analysis more explicit, specifying these two measures.

# Reviewer report: Reviewer response for version 2

**DOI:** 10.21956/hrbopenres.15422.r43949

**Published:** 2025-01-06

**Authors:** Meredith L. Rowe

**Affiliations:** 1 Harvard University, Cambridge, USA

Review of Study Protocol: “Evaluating a targeted selective speech, language, and communication intervention at scale – Protocol for the Happy Talk cluster randomised controlled trial” for HRB Open Research. 

    This is a proposed RCT to examine effects of the Happy Talk intervention for preschool educators and parents on preschool children’s language outcomes in Ireland. The literature review does a good job of making the case for the need (the low language skills in areas of social disadvantage) and for the approach (targeting teachers *and* parents) in this effectiveness trial in the community. 

 Is the rationale for, and objectives of, the study clearly described?

 Yes, for the most part, the research questions are clear and follow from the literature review.  My only question is about RQ4 and any hypotheses regarding the specific program features or contextual factors.  If there are no specific hypotheses here, and if this RQ is under-powered statistically, the authors might list this as an “exploratory analysis”.

 Is the study design appropriate for the research question?

    The longitudinal cluster randomized design is appropriate. I have a few small questions about the methods where more information could be provided.

 -are the randomly chosen 12 participants all going to be from lower-SES backgrounds? This seems important given the rationale

 -are the parents compensated for attending the sessions? What is the motivation for them and feasibility of them showing up based on prior work with this intervention? I see they are compensated when the child gets tested, but also for their own participation or do they opt in without this added incentive?

 -it might be important to see if the children in the classrooms of “communication champions” do better than those in other treatment classrooms

 -I wonder if a more nuanced measure of classroom language would be helpful in addition to the CLASS.  That is, if is it possible to audiotape a short “circle time” session for each participating teacher during the CLASS observation then this segment could be coded for teacher and classroom language use. For example, how many questions the teacher asks, how many children contribute to conversations, how extended the conversations are, etc.  This would be similar to the MRBCS but in the classroom context.  This is understandably a fair amount of work to record, and potentially transcribe and code, but it may result in a lot more variability in relevant measures than the CLASS and also in some insights as to the specific mechanisms of effects. 

 -Also, I would suggest potentially coding the child speech in the MRBCS samples as an additional measure of child language/vocabulary. 

 *these last two comments are based on the fact that many parent and preschool interventions have a hard time ‘moving the needle’ on standardized tests but are more likely to show effects on the day-to-day practices that are promoted. I worry that the vast majority of the measures here are more global and standardized and more subtle effects might be missed.  If coding videos is too time-consuming, maybe something like the Language Use Inventory (McNeill) that can be filled out by the teacher and the parent (of course there are bias issues here because of parent/teacher reporting). 

 Are sufficient details of the methods provided to allow replication by others?

 Yes, it appears so as long as the intervention itself is publicly available.

 Are the datasets clearly presented in a useable and accessible format?

 Data analysis and monitoring plans seem appropriate.

 -I am wondering if some children will potentially have a teacher who participated in the intervention 2 years and others just have 1 teacher and then a non-intervention teacher for the 1- year follow up?  If this is the case, this factor needs to be controlled/examined or potentially manipulated in classroom placement for the children randomly chosen to participate. 

 -the economic analysis is a strength of this study.

Is the study design appropriate for the research question?

Yes

Is the rationale for, and objectives of, the study clearly described?

Yes

Are sufficient details of the methods provided to allow replication by others?

Yes

Are the datasets clearly presented in a useable and accessible format?

Not applicable

Reviewer Expertise:

Language development, parenting, parent interventions, early childhood

I confirm that I have read this submission and believe that I have an appropriate level of expertise to confirm that it is of an acceptable scientific standard.

# Response

**Published:** 2025-01-23

**Authors:** Pauline Frizelle

**Affiliations:** Speech and Hearing Sciences, University College Cork, Cork, Ireland

Many thanks for your valuable feedback - we have addressed each point in turn

 Are the randomly chosen 12 participants all going to be from lower-SES backgrounds? This seems important given the rationale

**Response:** The profile of all settings is that they are in significantly disadvantaged areas and are designated DEIS pre/schools in Ireland (Delivering Equality and Opportunity in schools). We appreciate that there will be different degrees of disadvantage within a given setting, however, we will note these individual differences in the collection of our demographic data. We acknowledge that the same variation will exist within the control settings and therefore should not be a confounding variable.

 Are the parents compensated for attending the sessions? What is the motivation for them and feasibility of them showing up based on prior work with this intervention? I see they are compensated when the child gets tested, but also for their own participation or do they opt in without this added incentive?

**Response:** Parents are not compensated for attending the sessions, they opt in without additional incentives. In areas where Happy Talk is well established there is very high attendance as other parents/educators have spoken so positively about the programme and parents have an expectation that this will be part of their child’s education. In new areas (as is the case here) attendance tends to increase as the programme progresses. Parents become more committed as their confidence increases, and children request their parents to be present in the classroom, as they enjoy this aspect of the programme.

 it might be important to see if the children in the classrooms of “communication champions” do better than those in other treatment classrooms

**Response: **There will be a communication champion in all rooms in which there are children attending Happy Talk. We have clarified this in the text.

 I wonder if a more nuanced measure of classroom language would be helpful in addition to the CLASS.  That is, if is it possible to audiotape a short “circle time” session for each participating teacher during the CLASS observation then this segment could be coded for teacher and classroom language use. For example, how many questions the teacher asks, how many children contribute to conversations, how extended the conversations are, etc.  This would be similar to the MRBCS but in the classroom context.  This is understandably a fair amount of work to record, and potentially transcribe and code, but it may result in a lot more variability in relevant measures than the CLASS and also in some insights as to the specific mechanisms of effects. 

 -Also, I would suggest potentially coding the child speech in the MRBCS samples as an additional measure of child language/vocabulary. 

**Response: **We appreciate the reviewers comments with respect to capturing the day to day changes in child language and interaction. We are using the FOCUS-34 with the aim of capturing these functional changes at home and in school. Unfortunately time constraints and resources prevent us from recording and coding sessions in the classroom. There are also ethical issues as we would be actively recording children who have consented to receiving Happy Talk but have not consented to participate in the evaluation. We are cognisant of the burden of evaluation on families and for this reason, do not want to introduce any further outcome measures.

 We are reluctant to use the language produced during the MRBCS as a measure of child language /vocabulary for the reasons outlined in response to reviewer 1. Specifically, the videotape segment is intended to capture the use of language promoting strategies used by parents, is a very short sample, and subject to  significant variability due to differences in parental scaffolding. As such we would be concerned that it would not yield reliable MLU estimates due to the need for a considerable number of turns for such reliability. Also given that parents differ in their communication style, which affects the amount of language used by a child, this could be a confounding variable if we were to use the videos as estimates of language ability.

 *these last two comments are based on the fact that many parent and preschool interventions have a hard time ‘moving the needle’ on standardized tests but are more likely to show effects on the day-to-day practices that are promoted. I worry that the vast majority of the measures here are more global and standardized and more subtle effects might be missed.  If coding videos is too time-consuming, maybe something like the Language Use Inventory (McNeill) that can be filled out by the teacher and the parent (of course there are bias issues here because of parent/teacher reporting). 

 I am wondering if some children will potentially have a teacher who participated in the intervention 2 years and others just have 1 teacher and then a non-intervention teacher for the 1- year follow up?  If this is the case, this factor needs to be controlled/examined or potentially manipulated in classroom placement for the children randomly chosen to participate. 

**Response: **All educators and children will only participate in the intervention for one year. However, it is possible that some children will progress from an intervention preschool to a school that also participated in the intervention and where the teacher was trained in the Happy Talk programme. We will control for this factor.****

# Reviewer report: Reviewer response for version 1

**DOI:** 10.21956/hrbopenres.15338.r42876

**Published:** 2024-11-25

**Authors:** Heidi Feldman

**Affiliations:** 1 Stanford University, Stanford, California, USA

Background:  Social disadvantage is associated with delays and disorders in language development.  Previous studies have found that parent- or school-language enrichment programs are associated with slight to modest gains in child language outcomes.  This study protocol combines both parent and school language enrichment in an effort to maximize the positive impact.

 Study design: The proposed study is a large scale cluster randomised trial of a 12-week manualised intervention delivered in pre/school settings serving socially disadvantaged communities, in Ireland. 72 clusters receive the intervention (12 participants per cluster). Parents and pre/school staff engage in group training and coaching in the form of

 12 1-hour sessions for parents and four staff workshops for educators, over the course of the 8-month pre/school year.

**Is the rationale for, and objectives of, the study clearly described?**

 The study rests on reliable observations that children from disadvantaged backgrounds have disproportionately higher rates of early and persistent language disorders than children from advantaged backgrounds.  Language disorders predict important impacts for educational and occupational outcomes.  The scope of this problem makes addressing language delay in poor communities an appropriate public health target.  Further, given the high prevalence, a “tier 1” intervention, offered universally and preventatively, is highly appropriate.

 Previous linguistic and auditory intervention efforts directed to preschool educators have yielded variable findings and, at best, improvements with only modest effect sizes.  Professional literacy interventions have been somewhat better for reading outcomes with little generalization to language.  Similarly, preventive parent-directed interventions are also associated with small to moderate effect sizes. 

 Based on this review, the investigators plan to provide intervention in more than a single environment, aimed at both home and school.  Pilot data from Happy Talk in a single community of Ireland are promising, though other studies show no additional benefit.  The questions and hypotheses are clear and comprehensive. 

**Is the study design appropriate for the research question?**

 The study is a cluster-randomized controlled trial taking place over three years.  The intervention period is 8 months with immediate and delayed follow-up.  This design is appropriate and feasible as an effectiveness tiral.

 Participants will be targeted for recruitment from 4 Community Healthcare Organizations based on level of social disadvantage using a deprivation index.  These decisions are appropriate.  Exclusion criteria are reasonable.  The intervention is a manualized training and support programme delivered by Speech-Language Therapists (SLTs) to parents and early childhood educators. 

 One concerning feature about the protocol is that the child participants are aged 0-6.  This broad age range is problematic because the parent coaching for a 5–6-year-old would be exceedingly different from the appropriate strategies for a 1-2 year old.  Limiting the intervention to 18 or 24 months of age to 36 months of age might be a better test of the protocol.

 Another potentially concerning feature is that the parent intervention component requires 12 1-hour sessions in 4-week blocks and in 2 additional 30-minute units over the school year.  The investigators offer no rationale for this schedule.  There is no mention of engaging potential participants in the development of the curriculum and specifically in the duration or timing of these sessions.  The investigators plan a 2–3-week engagement phase with each school to build relationships, but parents are not included.  The 30 minute segments are in person at the center with direct coaching with parent and child.  When in the  course of the program will they be completed?  There is no discussion about the 12 1-hour sessions.  Are they in person?  Online?  Does this schedule work for working and busy parents?  There is also no discussion about the parents’ adherence to the plan.  The outcome measures mention maternal responsiveness.  Can fathers or other caregivers participate? 

 The Happy Talk intervention will be compared to treatment as usual (TAU).  This decision is very reasonable.

 The primary child outcome measures are the Pre-School Language Scales, 5 ^th^ edition and Focus on Outcomes of Communication Under 6, which is a parent report measure.  The use of a parent-report measure introduces potential bias because the intervention targets parents.  The secondary child measures, the PedsQL asks only about communication problems and is therefore likely to be highly insensitive in this setting.  The other secondary outcome, the Child Health Utility Instrument is yet another caregiver-completed questionnaire and has nothing to do with the interventions being offered.  Why should quality of life change?  Note that the educators are not providing any ratings as outcome measures.  Other potential ratings, such as a functional measure (ABBAS or Vineland) could be considered and these measures can be completed by parents and teachers..  There are two additional secondary measures—the Maternal Responsive  Behavior Coding scheme and the Classroom Assessment Scoring System.  Are fathers also invited to parent sessions?  Why use a maternal measure?  Rather than conceptualizing these measures as outcomes, they could be conceptualized as fidelity checks on the parents and teachers.  Then changes in them could be considered mediators of change in the child outcomes.  If parents or teachers are not changing as a function of the training, it is unlikely that the intervention will be successful.  Note that the maternal responsiveness measure will be based on a videotape segment.  Could the child's language in this segment be an outcome?

 Power analyses and the intention to treat rationale are well described.  There is no rationale for the HRQoL in the economic analyses.  Why should this assessment be sensitive to language interventions? 

 Another potential program is the randomization of sites scheme.  The investigators have not discussed the implications if a site that participated in Round 1 of the intervention is randomized to be control in Round 2.  Isn’t it likely that those trained educators will continue to use what they have learned in the next round? What about if classes contain siblings of previous participants?

 Economic analyses are planned.  The primary measures will be the standardised Health Related Quality of Life (HRQoL) questionnaires pre-, post- and at 12 months follow-up. HRQoL measures enable the calculation of Quality Adjusted Life Years (QALYs) as well as an investigation of the sensitivity of these measures with children. The investigators are wary of the plan.  It is unclear how QALYs relate to the improvement in language skills anticipated in this trial.  Could other methods be used, such as functional assessment (what is the value of increasing a child’s standard score on a functional measure in terms of later outcomes?)

**Are sufficient details of the methods provided to allow replication by others?**

 The protocol is very detailed.  The curricula are not included but other than that, the study is reproducible.

 Are the datasets clearly presented in a useable and accessible format?  Not applicable.

 In summary, this ambitious trial may have important public health implications for disadvantaged children in Ireland and beyond.  The study could be strengthened with adjustments in the planned methods:

1. Narrow the age range of participants.  Infants less than one and children over about 4 need different types of language input than toddlers and young preschoolers. 
2. Engage parents in developing their intervention.  Check on their ability to attend 12 1-hour sessions. 
3. Consider the secondary measures as fidelity checks on parents and educators and adjust the statistical analyses accordingly. 
4. Secondary child outcomes are weak.  They rely on parent reports and are not sensitive to advances, but rather child weaknesses.  No educator ratings are planned.  Why not use a functional measure, such as the Vineland.  Alternatively, the investigators could code child language from the MRBC videos.
5. Consider dropping the health-related quality-of-life measure as it has no relation to the intervention.
6. Rethink the economic analyses based on QALYs and HRQOL. 

Is the study design appropriate for the research question?

Yes

Is the rationale for, and objectives of, the study clearly described?

Yes

Are sufficient details of the methods provided to allow replication by others?

Yes

Are the datasets clearly presented in a useable and accessible format?

Not applicable

Reviewer Expertise:

language development, developmental disabilities, pediatrics, developmental neuroscience

I confirm that I have read this submission and believe that I have an appropriate level of expertise to confirm that it is of an acceptable scientific standard, however I have significant reservations, as outlined above.

# Response

**Published:** 2024-12-05

**Authors:** Pauline Frizelle

**Affiliations:** Speech and Hearing Sciences, University College Cork, Cork, Ireland

We thank the reviewer for her positive evaluation of the protocol, and for the suggestions she has made to improve it. We document here how we have responded to each point.

 A point of clarification - The reviewer notes that participants will be targeted for recruitment from 4 Community Healthcare Organizations (CHOs) based on level of social disadvantage using a deprivation index. Participants will be recruited from 8  Community Healthcare Organizations (4 each year).

  

1. Narrow the age range of participants.  Infants less than one and children over about 4 need different types of language input than toddlers and young preschoolers. 

**Response** - The reviewer notes that it is concerning that child participants span a very broad age range i.e. 0-6.  For clarification, the overall Happy Talk programme provides services for children 0-6 years, but this evaluation is focussed on the preschool programme only, which ranges from 2;10 months to a maximum of 6 years. However the majority of children fall within the 3 – 5 year age range.   

  

1. Engage parents in developing their intervention.  Check on their ability to attend 12 1-hour sessions. When in the  course of the program will they be completed?  There is no discussion about the 12 1-hour sessions.  Are they in person?  Online? 

**Response** – This intervention has been implemented in one area of Ireland (to over 80 settings) for in excess of 11 years. The feasibility of the programme with parents living in socially disadvantaged areas has long been established. Feedback from parents and early years educators has been embedded in the programme since it was first developed. We realise that there will always be variability in parental attendance particularly when a programme is not yet established in an area. We had specified that the 12 1 hours sessions took place in 4 week blocks, 1 between September and December, 1 between January and March and 1 between April and June. We have clarified that they are in person.

  

1. Consider the secondary measures as fidelity checks on parents and educators and adjust the statistical analyses accordingly. Rather than conceptualizing these measures as outcomes, they could be conceptualized as fidelity checks on the parents and teachers.  Then changes in them could be considered mediators of change in the child outcomes.  If parents or teachers are not changing as a function of the training, it is unlikely that the intervention will be successful.  Note that the maternal responsiveness measure will be based on a videotape segment.  Could the child's language in this segment be an outcome?

**Response: **In line with the reviewer and our logic model/ theory of change, we consider both the parent and educator outcome measures as  mediators of change in the child outcomes. In our pilot trial we were unable to show significant change in our parent group and did not have a sufficient number of settings to explore the impact of potential changes in the preschool environment. Despite this, the intervention had a large effect on the participating children. However, we will add an exploratory set of models to the statistical analysis plan aimed at evaluating heterogeneity in treatment effects on child outcomes as a function of parent and educator outcomes.

 In addition, parents and teachers will be completing their own separate fidelity checks and 20% of the intervention sessions will also be coded for fidelity against the manual. The videotape segment is intended to capture the use of language promoting strategies used by parents, is a very short sample, and subject to  significant variability due to differences in parental scaffolding. As such we would be concerned that it would not yield reliable MLU estimates due to the need for a considerable number of turns for such reliability. Also given that parents differ in their communication style, which affects the amount of language used by a child, this could be a confounding variable if we were to use the videos as estimates of language ability.

  

1. Secondary child outcomes are weak.  They rely on parent reports and are not sensitive to advances, but rather child weaknesses.  No educator ratings are planned.  Why not use a functional measure, such as the Vineland.  Alternatively, the investigators could code child language from the MRBC videos.

**Response:** We are capturing changes in child language through our primary outcomes measures, a standardized composite language measure (the PLS- 5) and a functional measure  (the FOCUS- 34). Changes in parent behaviour are captured through the MRBCS, - an observation schedule shown to capture key characteristics associated with language growth over time (Down et al., 2015; Levickis et al., 2014; Smith et al., 2019) . The secondary outcome measures are as the reviewer suggests parent proxy measures, however literature suggests that parental measures are considered reliable and with acceptable concurrent validity (Feldman et al., 2005). The CLASS  secondary outcome measure is a reliable measure which will allow us to assess change in the quality of interactions between teachers and students in the classroom, this is an aspect of our logic model which we deem important to capture. 

 The Vineland is a measure of overall cognition rather than one that focuses specifically on language.

 We have not included educator ratings of children, as the staff working with children are not always consistent and they are working with large groups of children, only some of which are included in the evaluation.  We do not want them to focus their attention/prioritise  those in the evaluation over and above those who are not.   

  

1. Consider dropping the health-related quality-of-life measure as it has no relation to the intervention.

**Response** – We agree that the HRQL measures are not ideal and may not be  sensitive to the changes that we expect as a result of our intervention, this was the case in our pilot trial. However, our pilot trial assessments took place over a short time period and it is not recommended to complete these measures over a period shorter than a year. Improvement in language outcomes are associated with overall well-being and these HRQL measures do ask about communication, which is at the core of our intervention. We do not have other measures at our disposal that can generate QALYs and are therefore suitable for a primary cost effectiveness analysis.

   

1. Rethink the economic analyses based on QALYs and HRQOL. 

**Response** - We have given these measures considerable thought and in discussion with our health economist had decided to include them as they are not onerous to complete and there are currently no better alternatives. We have however also included the FOCUS – 34 as our functional measure and the PLS-5 scores will be used in our secondary cost effectiveness analysis.  Our pilot trial included a value of information analysis, and indicated that there was value in collecting further information in a large scale trial.

  

1. The randomization of sites scheme.  The investigators have not discussed the implications if a site that participated in Round 1 of the intervention is randomized to be control in Round 2.  Isn’t it likely that those trained educators will continue to use what they have learned in the next round? What about if classes contain siblings of previous participants?

**Response**- Round 2 will involve the recruitment of 4 additional sites (in different areas) and will have no relationship with the sites recruited  in round 1.

 Down, K., Levickis, P., Hudson, S., Nicholls, R. and Wake, M. (2015), Measuring maternal responsiveness. Child Care Health Dev, 41: 329-333. https://doi.org/10.1111/cch.12174

 Feldman HM, Dale PS, Campbell TF, Colborn DK, Kurs-Lasky M, Rockette HE, Paradise JL. Concurrent and predictive validity of parent reports of child language at ages 2 and 3 years. Child Dev. 2005 Jul-Aug;76(4):856-68. doi: 10.1111/j.1467-8624.2005.00882.x. PMID: 16026501; PMCID: PMC1350485.

 Levickis, Penny PhD ^*,†,‡^; Reilly, Sheena PhD ^*,‡^; Girolametto, Luigi PhD ^§^; Ukoumunne, Obioha C. PhD ^‖^; Wake, Melissa MD ^*,†,‡^. Maternal Behaviors Promoting Language Acquisition in Slow-to-Talk Toddlers: Prospective Community-based Study. Journal of Developmental & Behavioral Pediatrics 35(4):p 274-281, May 2014. | DOI: 10.1097/DBP.0000000000000056

 Smith, J., Levickis, P., Eadie, T., Bretherton, L., Conway, L., & Goldfeld, S. (2019). Associations between early maternal behaviours and child language at 36 months in a cohort experiencing adversity. *International journal of language & communication disorders*, *54*(1), 110–122. https://doi.org/10.1111/1460-6984.12435
